# 

**Published:** 1866-04

**Authors:** 


For sale by W. B. Keen & Co., 148 Lake St.

We have also received the following valuable and interesting works, namely:

Lectures on Epilepsy, Pain, Paralysis, and certain other Disorders of the Nervous System. By Charles Bland Radcliffe, M.D., Fellow of the Royal College of Physicians of London; Physician to the Westminster

Hospital, and to the National Hospital for the Paralyzed and Epileptic, etc. Philadelphia: Lindsay & Blakiston. 1866.

A small-sized octavo of 280 pages.

---

The Physiology of Man; Designed to Represent the Existing State of Physiological Science, as Applied to the Functions of the Human Body. By Austin Flint, Jr., M.D., Prof. Physiology and Microscopy, in the Bellevue Hospital Medical College, New York, and in the Long Island College Hospital; Fellow of the New York Academy of Medicine; Microscopist to Bellevue Hospital. New York: D. Appleton & Co. 1866.

A full-sized octavo volume of 502 pages, embracing the discussion of the following topics:—Introduction; the Blood; Circulation; and Respiration.

For sale by S. C. Griggs & Co., Lake St. Price $4.50.

---

A Practical Treatise on Urinary and Renal Diseases, including Uri-Deposits. Illustrated by numerous Cases and Engravings. By William Roberts, M.D., F.R.C., London; Physician to the Manchester Royal Infirmary; Lecturer on Medicine in the Manchester School of Medicine. Philadelphia: Henry C. Lea. 1866. Octavo, pp. 516.

For sale by W. B. Keen & Co., 148 Lake St.

---

Extract from an Unpublished Essay on Physical Force. By Louis Mackall, M.D. Washington. 1865.

An Essay on the Life in Nature. By Louis Mackall, M.D. Washington. 1865.

An Essay on the Law of Muscular Action. By Louis Mackall, M.D. Washington. 1865.

The last three essays are pamphlets, varying from 16 to 34 pages.

### CHICAGO MEDICAL EXAMINER.

**N. S. DAVIS, M.D., EDITOR.**

A MONTHLY JOURNAL

DEVOTED TO THE

EDUCATIONAL, SCIENTIFIC, AND PRACTICAL INTERESTS

OF THE

MEDICAL PROFESSION.

The EXAMINER will be issued during the first week of each month, commencing with January, 1860. Each number will contain 64 pages of reading matter, the greater part of which will be filled with such contents as will directly aid the practitioner in the daily practical duties of his profession.

To secure this object fully, we shall give, in each number in addition to ordinary original articles, and selections on practical subjects, a faithful report of many of the more interesting cases presented at the Hospitals and College Cliniques. While aiming, however, to make the EXAMINER eminently practical, we shall not neglect either scientific, social, or educational interests of the profession. It will not be the special organ of any one institution, society, or clique; but its columns will be open for well-written articles from any respectable member of the profession, on all topics legitimately within the domain of medical literature, science, and education.

Terms, $3.00 per annum, invariably in advance.

